# Sparse Estimation of Resting-State Effective Connectivity From fMRI Cross-Spectra

**DOI:** 10.3389/fnins.2018.00287

**Published:** 2018-05-08

**Authors:** Carolin Lennartz, Jonathan Schiefer, Stefan Rotter, Jürgen Hennig, Pierre LeVan

**Affiliations:** ^1^Department of Radiology, Medical Physics, Medical Center, University of Freiburg, Faculty of Medicine, University of Freiburg, Freiburg, Germany; ^2^BrainLinks-BrainTools Cluster of Excellence, University of Freiburg, Freiburg, Germany; ^3^Bernstein Center Freiburg & Faculty of Biology, University of Freiburg, Freiburg, Germany

**Keywords:** effective connectivity, functional connectivity, structural connectivity, fMRI, resting state, correlation

## Abstract

In functional magnetic resonance imaging (fMRI), functional connectivity is conventionally characterized by correlations between fMRI time series, which are intrinsically undirected measures of connectivity. Yet, some information about the directionality of network connections can nevertheless be extracted from the matrix of pairwise temporal correlations between all considered time series, when expressed in the frequency-domain as a cross-spectral density matrix. Using a sparsity prior, it then becomes possible to determine a unique directed network topology that best explains the observed undirected correlations, without having to rely on temporal precedence relationships that may not be valid in fMRI. Applying this method on simulated data with 100 nodes yielded excellent retrieval of the underlying directed networks under a wide variety of conditions. Importantly, the method did not depend on temporal precedence to establish directionality, thus reducing susceptibility to hemodynamic variability. The computational efficiency of the algorithm was sufficient to enable whole-brain estimations, thus circumventing the problem of missing nodes that otherwise occurs in partial-brain analyses. Applying the method to real resting-state fMRI data acquired with a high temporal resolution, the inferred networks showed good consistency with structural connectivity obtained from diffusion tractography in the same subjects. Interestingly, this agreement could also be seen when considering high-frequency rather than low-frequency connectivity (average correlation: *r* = 0.26 for *f* < 0.3 Hz, *r* = 0.43 for 0.3 < *f* < 5 Hz). Moreover, this concordance was significantly better (*p* < 0.05) than for networks obtained with conventional functional connectivity based on correlations (average correlation *r* = 0.18). The presented methodology thus appears to be well-suited for fMRI, particularly given its lack of explicit dependence on temporal lag structure, and is readily applicable to whole-brain effective connectivity estimation.

## Introduction

In recent years, brain connectivity analysis of functional magnetic resonance imaging (fMRI) data has become of high interest, particularly as many diseases such as Alzheimer's and epilepsy are now understood as cerebral network malfunctions (Fisher et al., 2017; Ofer et al., 2018). Functional MRI is a non-invasive method that can monitor whole-brain functional activity. In resting state fMRI (rs-fMRI), relationships between intrinsic fluctuations across multiple brain areas are analyzed, giving rise to the concept of the brain as a network (Biswal et al., 1995). For connectivity analyses, it is common to consider *functional connectivity* (FC), which is retrieved by calculating the correlation between the time series from different brain areas. However, this approach exhibits some limitations (Stephan, 2004; Petersen and Sporns, 2015) as it yields only symmetric connections, with no information on their direction. Moreover, conventional approaches using raw correlations may reflect indirect connections between brain areas that are not actually directly linked.

Of great interest are methods that retrieve information about the existence and direction of connections, and which can rule out indirect connections. The *effective connectivity* (EC) describes “the influence one neural system exerts over another” (Friston, 1994), or as Aertsen and Preißl (1991) put it, EC is “the simplest possible circuit diagram that would replicate the observed timing relations” between observed responses and therefore describes directed connectivity. Although several different approaches have been suggested to estimate EC, the most widely used methods for fMRI data are Granger Causality (Bressler and Seth, 2011) and Dynamic Causal Modeling (Friston et al., 2003).

Granger causality (GC) exploits temporal precedence between two time series to estimate the direction of the connections. It exists both for time domain (Geweke, 1982) and frequency domain (Geweke, 1984; Baccalá and Sameshima, 2001) data. In order to estimate GC, usually a vector autoregressive process is fit to the data, which can be problematic as fMRI signals typically have a temporal resolution of 1–3 s (Lin et al., 2014), whereas characteristic time scales of neuronal processes are in the order of tens to hundreds of milliseconds. Moreover, temporal relationships between cerebral areas are confounded by the spatial variability of the hemodynamic response function (Handwerker et al., 2004). Although MR acquisition sequences with faster temporal resolutions are becoming increasingly common (Feinberg et al., 2010; Posse et al., 2012; Akin et al., 2017; LeVan et al., 2017), neuronal processes still undergo considerable downsampling in fMRI time series, affecting the reliability of GC estimates (Seth et al., 2013; Friston et al., 2014a).

Dynamic causal modeling (DCM) is a framework fitting differential equations to the fMRI data to yield parameters for the strength of connections, as well as the strength of the influence of external stimuli on connectivity. In the classical deterministic DCM, but also stochastic DCM (Li et al., 2011), the neuronal activity underlying the BOLD response is determined by a bilinear model, whereas the hemodynamic response is estimated using the Balloon model (Buxton et al., 1998; Friston et al., 2000). DCM requires to define a model a priori to test different specific hypotheses, which can then be compared via Bayesian model comparison (Penny et al., 2004, 2010; Penny, 2012). While the classical or stochastic DCM is only suited for task data with known input functions, a DCM for resting state data was developed recently (Friston et al., 2014b), which fits a model to the cross-spectrum of the data. However, due to the computational complexity of the differential equations, DCM is not suited for whole-brain connectivity analysis. Furthermore, with growing size of the models, non-identifiability becomes an issue of increasing severity (Arand et al., 2015; Frässle et al., 2015).

Aiming to overcome some of the issues outlined above, we present a methodology to estimate the EC from the frequency-domain cross-spectral density (CSD). Similar to the GC approach, the fMRI data are expressed as a multivariate autoregressive process, which is computationally suitable to model a large number of nodes in whole-brain datasets. However, unlike GC or other similar lag-based methods (see Smith et al., 2011 for a review of several such methods), we do not make use of temporal precedence to define the directionality of the estimated connections, thus partially circumventing hemodynamic confounds on the lag structure of fMRI time series. Rather, a directed and potentially asymmetric network is estimated in such a way as to explain the observed cross-spectral density matrix. As temporal precedence is not enforced, this is an underdetermined problem with a potentially infinite number of solutions, so we additionally constrain the network to have the smallest number of non-zero connections using an L1 minimization on the entries of the connectivity matrix.

One issue when validating EC estimation in real fMRI data is the lack of an ideal ground truth. One popular approach is to use information from structural connectivity (SC), which can be estimated using diffusion-tensor imaging (DTI). Using tractography algorithms (Wedeen et al., 2008; Reisert et al., 2013) on the DTI data, the white matter tracts forming connections between different regions can be reconstructed and the number of “fibers” (streamlines) can be used as a proxy for the strength of these connections. SC is commonly used to constrain the estimation (Gilson et al., 2016; Crimi et al., 2017; Dang et al., 2018), or may be used independently to validate the estimated EC (Uddin et al., 2011; Bringmann et al., 2013). However, there are also clear limitations to such approaches, as EC is dynamic and potentially brain-state-dependent as opposed to static SC. As such, while SC is often used as a proxy for connectivity and reasonable agreement is found between FC and SC (Li et al., 2012; Finger et al., 2016), we should not expect complete concordance between SC and EC, although the two measures should still be consistent with each other.

In the remaining sections we will briefly explain the mathematical background and implementation of the method. In a simulation study, the influence of several parameters on the estimation will be analyzed. Finally, we will apply the methodology to real resting-state fMRI data. In the absence of ideal validation measures in real data, the consistency of the estimated effective connectivity with the structural connectivity from white matter tracts will then be assessed.

## Materials and methods

### Methodology

#### Mathematical and algorithmic background

We consider networks of *n* interconnected neuronal populations. Each population is characterized by neuronal activity *y*_*i*_(*t*) with *i* ∈ [1, *n*]. Similar to the GC framework, we assume that the neuronal activity follows a generic multivariate autoregressive process

(1) $$\begin{array}{l} y\left(t\right)=x\left(t\right)+\int_{-\infty }^{t}G\left(t-u\right)y\left(u\right)du\\ =x\left(t\right)+G*y\left(t\right) \end{array}$$

which describes how the neuronal activity $y\left(t\right)={\left[y_{1}\left(t\right),y_{2}\left(t\right),\ldots ,y_{n}\left(t\right)\right]}^{T}$at time point *t* in each population depends on the driving “noise” (or external stimuli) ***x***(*t*) and the activity in other populations with time lag *u* via the linear coupling kernel ***G***(*t*), where ***G***_*ij*_(*t*) describes the influence of node *j* on node *i*. The coupling can be described by a convolution (“^*^”) of ***G***(*t*) with the neuronal activity ***y***(*t*).

Now, in the GC framework, a causal system would then be assumed by additionally setting ***G***(*t*) = 0 for negative time lags *t* < 0, and the remaining coefficients of ***G*** could then be fitted by linear regression, with non-zero coefficients indicative of a directed influence of one node on another inferred from their temporal precedence relationship (Goebel et al., 2003; Duggento et al., 2016). This approach can also been extended to support non-linear interactions (Harrison et al., 2003) and couplings that are dynamically fluctuating over time (Smith et al., 2013; Park et al., 2017; Samdin et al., 2017). However, as outlined above, fMRI only indirectly measures neuronal activity in the form of the BOLD signal, yielding low temporal resolutions and spatially variable lag structure that confound GC estimates (Deshpande et al., 2009; Rogers et al., 2010).

Circumventing these issues, we deviate from the GC framework and do not enforce the causality of ***G*** and thus do not rely on temporal precedence relationships to identify directed connections. Rather, we rely on the observation that cross-correlations, which are symmetric and thus undirected, nevertheless contain information about the underlying directed (and thus potentially asymmetric) network, notably the presence of so-called “collider” structures (Ramsey et al., 2010; Pernice and Rotter, 2013). Based on frequency-domain cross-spectra, we thus estimate a directed network independently of temporal precedence relationships.

Applying the Fourier transform to Equation 1, we get $\hat{y}\left(f\right)=\hat{x}\left(f\right)+\hat{G}\left(f\right)\hat{y}\left(f\right)$, where $\hat{.}$ depicts the Fourier transform of the respective variable. Assuming that both the intrinsic noise ***x***(*t*) and the neuronal activity ***y***(*t*) are stationary stochastic processes, the cross-spectral density can be derived (Hawkes, 1971; Pernice and Rotter, 2013):

(2) $$\langle \hat{y}(f){\hat{y}}^{*}(f)\rangle =\hat{C}\left(f\right)={\left[1-\hat{G}\left(f\right)\right]}^{-1}\hat{X}\left(f\right){\left[1-{\hat{G}}^{*}\left(f\right)\right]}^{-1}$$

$\hat{G}\left(f\right)$ is the frequency-dependent coupling matrix, 1 the identity matrix, $\hat{X}\left(f\right)=<\hat{x}\left(f\right){\hat{x}}^{*}\left(f\right)>$ depends on the driving noise, and <,> is the time expectation operator. Noise is assumed to be independent and Gaussian, so that $\hat{X}\left(f\right)$ is a diagonal matrix of (unknown) noise variances.

We are ultimately interested in recovering the effective connectivity $\hat{G}\left(f\right)$ of the network of neuronal populations, given only $\hat{C}\left(f\right)$, the cross-spectral density matrix of the measured activity ***y***(*t*). Taking the inverse of the CSD [2] we get

(3) $${\hat{C}}^{-1}(f)=\;1-{\hat{G}}^{*}\left(f\right)\;{\hat{X}}^{-1}\left(f\right)\left[1-\hat{G}\left(f\right)\right]=\;B^{*}(f)B(f)$$

with $B(f)={\sqrt{\hat{X}\left(f\right)}}^{-1}\left[1-\hat{G}\left(f\right)\right]$.

Given an estimate of ***B*(***f***)**, the coupling matrix

(4) $$\hat{G}\left(f\right)=1-\sqrt{\hat{X}\left(f\right)}\;B(f)$$

can be estimated only up to a positive factor $\sqrt{\hat{X}\left(f\right)}$ as the covariance of the intrinsic noise is not known. The matrix ***B*(***f*), nonetheless, gives information about strength, sign and direction of connections since $\hat{X}\left(f\right)$ is diagonal, although it may affect the scaling of the estimated weights (For better readability the dependency of the variables on the frequency is dropped from here on).

The computation of ***B*** from the CSD is, however, not straight forward, because it is not uniquely defined: Many different network topologies can give rise to the same CSD. More precisely, the decomposition of the CSD is only defined up to an arbitrary unitary transformation ***U*** since

(5) $$\begin{array}{l} {\hat{C}}^{-1}=B^{*}B=B^{*}U^{*}UB. \end{array}$$

To resolve this ambiguity, we assume that the network formed by the neuronal populations is sparse, which entails minimizing the L1-norm of the entries of the matrix ***UB***. The corresponding cost function is

(6) $$\begin{array}{l} \Gamma \left(UB_{0}\right)=||UB_{0}|{|}_{1}=\sum_{i\neq j}|{\left(UB_{0}\right)}_{ij}|=\sum_{i\neq j}|\sum_{k}U_{ik}B_{0,kj}| \end{array}$$

where ***B***_0_ is the initial guess of the decomposition. So the problem is to find the unitary transform ***U*** minimizing the cost function Γ(***UB***_0_) (Pernice and Rotter, 2013; Schiefer and Rotter, 2016)

$$\begin{array}{l} {\text{argmin}}_{U}\Gamma \left(UB_{0}\right)\\ s.t.{UU}^{*}=1 \end{array}$$

Geometrically this optimization can be viewed as a complex rotation of the cross-spectral density matrix, which can be implemented using a conjugate gradient descent algorithm (Abrudan et al., 2008, 2009).

For the estimation of the effective connectivity, each frequency bin of the CSD is treated separately, leading to a frequency-dependent connectivity. As starting point ***B***_0_ for the estimation, the positive definite matrix square root of ${\hat{C}}^{-1}\left(f\right)$ is chosen.

#### Threshold from null distribution

To exclude statistically non-significant connections in the estimated connectivity matrix, a threshold for each frequency is derived from a null distribution. The null distribution is computed by first splitting the time series into equal segments, shuffling the segments randomly and differently for each time series, and finally computing the CSD on the shuffled segments using Welch's method

(7) $$\begin{array}{l} CSD_{ij,\;null}\left(f\right)=\frac{1}{n}\sum_{k=1}^{n}{\hat{y}}_{ik}\left(f\right)\cdot {\hat{y}}_{j\sigma \left(k\right)}\left(f\right)\; \end{array}$$

where σ(*k*) is a permutation mapping. The shuffling will only affect the cross-spectra, while the power spectra (diagonal of the CSD matrix) will be preserved. Calculating connectivities from this null CSD and assuming that these values are to a great extent independent, a distribution of effective connectivity values is then derived. The 2.5 and 97.5% quantiles then yield *p* < 0.05 lower and upper thresholds for the connectivity matrices.

#### Confidence intervals from bootstrapping

We also derive confidence intervals for the connection strengths using bootstrapping. This could also be used to exclude connections that include zero in their confidence interval.

To derive the confidence intervals, the time series are again split into segments which are Fourier transformed. These segments are then drawn randomly with replacement and the CSD is calculated with the order of the segments kept identical for each time series

(8) $$\begin{array}{l} \text{CS}{\text{D}}_{\text{ij},\text{ bootstrap}}\left(\text{f}\right)=\frac{1}{\text{n}}\sum_{\text{k}=1}^{\text{n}}{\hat{y}}_{\text{i}\gamma \left(\text{k}\right)}\left(\text{f}\right)\cdot {\hat{y}}_{\text{j}\gamma \left(\text{k}\right)}\left(\text{f}\right)\; \end{array}$$

where Γ(*k*) is a permutation with replacement, which is the same for both time series ${\hat{y}}_{i}$ and ${\hat{y}}_{j}$. Calculating several bootstrap CSDs and estimating the connectivity thereof, a distribution of connection strength can be derived for each connection. Assuming an asymptotic Gaussian distribution of the parameter values, confidence intervals can then be determined.

### Data acquisition

#### fMRI acquisition and pre-processing

For experiments with real data, all measurements were performed on a 3 T Prisma scanner (Siemens Healthineers, Erlangen, Germany). Seven healthy volunteers, five male and two female in the age between 18 and 49, underwent a 20 min resting-state fMRI scan using the MREG sequence (Hugger et al., 2011; Assländer et al., 2013) with *TR* = 0.1 s, *TE* = 36 ms, *FA* = 25°, 64 × 64 × 50 matrix and 3 mm isotropic voxel size. T1-weighted MPRAGE images (*TR* = 2,000 ms, *TE* = 4.11 ms, FOV = 256 mm, 256 × 256 matrix, 160 sagittal slices, 1 mm slice thickness) were acquired for anatomic reference. Cardiac and respiratory fluctuations were additionally recorded with ECG and abdominal breathing band from the scanner's physiological monitoring unit. This study was approved by the Ethics Committee of the University Medical Center Freiburg. All subjects gave written informed consent in accordance with the Declaration of Helsinki. The data is available via the Open Science Framework repository (https://osf.io/52mf4/).

The fMRI data was motion corrected using FSL. Physiological noise correction was conducted with RETROICOR (Glover et al., 2000). The fMRI data sets were registered to their corresponding T1-images, which were in turn registered to MNI space. The registered fMRI data sets were parcellated according to the AAL-atlas and mean activity was calculated within each atlas region, excluding the cerebellum. The CSD was calculated for each dataset using Welch's method with a Hanning window with 50% overlap between windows.

To ensure that the CSD has full rank to be invertible, the number of frequency bins needs to be smaller than the degrees of freedom, i.e., the number of Fast Fourier Transform bins $NFFT\;<\frac{\#\;time\;points}{\#\;nodes}$. As the convolution with the HRF further reduces the degrees of freedom, the number of frequency bins was further decreased to the next lower power of two. Finally, the effective connectivity was extracted from the CSD for each frequency by sparse optimization as described in section Mathematical and Algorithmic Background.

#### DTI acquisition and pre-processing

In the absence of a gold standard for validation, a comparison with structural connectivity was performed. Thus, diffusion-weighted data was also acquired during the MRI sessions (61 diffusion directions, *TR* = 6.6 s, *TE* = 80 ms, *b* = 1,000 s/mm^2^, 60 slices, 2 mm isotropic voxel size). Using a global fiber tractography algorithm (Reisert et al., 2013) the structural connectivity could be extracted by counting streamlines connecting each pair of brain regions. Fiber endpoints lying in brain areas not covered by a region in the AAL atlas were reassigned to the nearest AAL area.

A summary SC matrix across all subjects was also generated from the individual SC matrices by considering connections existing in at least two thirds of the subjects.

#### Simulation study

As a proof of principle we first applied the method to simulated fMRI data. Moreover, we investigated the influence of several parameters on the estimation of the effective connectivity.

For this purpose, a vector autoregressive process of order 50 (VAR[50]) corresponding to a maximum conduction delay of 5 s was used with an additional contemporaneous term to model instantaneous self-excitation effects in each node and driving noise ***e***(*t*):

$$\begin{array}{l} y\left(t\right)=\overset{50}{\underset{p=0}{\sum }}G\left(p\right)y\left(t-p\right)+e\left(t\right) \end{array}$$

where **G**(*p* = 0) is the identity matrix. To simulate the oscillatory nature of resting-state fMRI data, the intrinsic activity in each node of the network was modeled as a noisy superposition of harmonic oscillations with different phases and frequencies. We chose a connection probability of each pair of nodes of 15% to model a sparse network. The coupling matrices **G**(*p*), which were modeled using random Erdős-Rényi networks, were the same for every lag p, however, they decreased in strength following a logistic decay. As a last step, the time series were convolved with the canonical hemodynamic response function (HRF) to simulate BOLD responses.

Functional magnetic resonance imaging (fMRI) observational noise is made up of several noise sources like scanner, physiological and temporal noise. The scanner noise is inherent in all fMRI data and can be modeled by Gaussian white noise (Gudbjartsson and Patz, 1995; Welvaert et al., 2011) given sufficient signal to noise ratio (SNR). Structured physiological noise corresponds to respiratory and cardiac oscillations. As we have a relatively high temporal resolution in our data, we assumed that most of the physiological noise could be filtered out. Therefore, it was not modeled in the simulated data. Temporal noise accounts for various sources of fluctuations with temporal autocorrelation (Purdon and Weisskoff, 1998). This was modeled using an AR[1] with coupling strength 0.5. Thus, observational noise with both white “scanner” noise and “temporal” noise in the form of an AR[1] were added.

We performed 20 simulations with 100 nodes. For each simulation the same network connectivity was used, but with different noise realizations. Furthermore, because a fast fMRI sequence with a *TR* = 0.1 s was used for the real fMRI measurements, our simulations of the VAR-process and the convolution with the HRF were performed with this temporal resolution with 51,000 data points corresponding to a 85 min measurement.

#### Connectivity analysis

Following the procedure outlined in section Methodology, the connectivity matrices were estimated for each frequency *f* from the CSD. The raw estimated connectivities were used directly for the analysis of the influence of parameters such as length of the time series. For the final performance analysis, however, the connectivities were additionally thresholded using the previously described null distribution and confidence intervals for each frequency bin. For the null distribution (Equation 7), as the “connections” in the null connectivity matrices are independent, only 10 cross-spectral density matrices were calculated per frequency and all derived connections were pooled to build a null distribution with 100,000 entries in the histogram. For the confidence intervals (Equation 8), 1,000 bootstrapped CSDs were calculated for each frequency.

##### Simulation study

Various simulations were performed to investigate the influence of the following parameters:

##### Length of the time series

Lengths were varied between 3,000 and 51,000 data points in steps of 6,000 (= 10 min). Furthermore, the SNR (ratio of signal variance to noise variance) was varied between 1 and 5.

##### Type of observational noise

Data were simulated with either pure white noise, a more realistic temporally correlated noise, or a mixture of both

$$\begin{array}{l} e=a\left(1\right)\cdot e_{white}+a\left(2\right)\cdot e_{temp} \end{array}$$

where a (1) is 0.3 and a (2) is 0.7. We again varied the SNR between 1 and 5 and used time series lengths of 20,000 (~35 min) or 40,000 (~70 min) data points.

##### Number of observed nodes

If connectivity analyses are performed in a given subnetwork of interest rather than the whole brain, hidden nodes exerting an influence on the observed nodes might yield erroneous results. To investigate this, connectivity estimations were performed within various fractions of the whole network in steps of 10 nodes, using either 20,000 or 40,000 data points and an SNR of 5.

##### Hemodynamic variability

In order to investigate the sensitivity of the estimation to hemodynamic variability, the activity at each node was subjected to a different random HRF instead of the canonical HRF. The random HRFs were generated using a double-gamma model, where the onset times of each gamma function was varied by up to 5 s, while the dispersion and amplitude parameters were varied by a factor of up to 5.

Finally, the recovery of an “average” group-level network was investigated using the 20 simulated realizations of the same network with an SNR of 5 and a length of 40,000 data points. After using the null distribution and confidence intervals to remove non-significant connections, averaging the resulting networks was not possible since connections did not necessarily exist in all datasets, so the mean network was defined as connections existing in at least half of the 20 data sets.

The comparison of the estimated EC with the true connectivity was done using correlation between the connectivity matrices. However, the correlation could be high even if many erroneous connections were detected, as long as all true connections are also found. Therefore, the area under the receiver-operator characteristic (ROC) curve (AUC) was also calculated to gain information about sensitivity of the estimation, where the ROC curve was obtained by varying the threshold on the estimated EC matrices.

##### fMRI data

For real fMRI data, the effective connectivity matrices were also derived for each frequency and non-significant connections were removed by calculating the threshold from the null distribution and deriving the confidence intervals.

To analyse the variability of the derived networks over subjects, the correlation and area under the ROC curve were calculated for the EC and SC networks between all subjects for each frequency. To compute an average connectivity over all subjects, only connections which existed in at least half of the subjects (4 in this case) were kept in the connectivity matrix. While the true underlying connectivity is not known, consistency was nevertheless assessed between the estimated EC and SC from DTI. However, because SC is symmetric, the estimated EC networks were first “mirrored” by adding the transposed connectivity matrix to the normal connectivity matrix. It was thus not possible to strictly validate the directionality of the estimated connections; in the absence of suitable gold standard, this approach is nevertheless expected to provide a limited degree of validation in real data.

Furthermore, EC was also compared to standard functional connectivity, represented by the raw cross-spectral density between time series from the various regions of interest. The correlation and AUC values for the comparison between EC and SC and FC and SC were calculated for each frequency and each subject. Furthermore, the agreement of SC and EC/FC was compared by determining the percentage of connections fulfilling each of the three following cases: (1) The connection is present in both SC and EC/FC, (2) the connection is present in EC/FC, but not in SC, and (3) the connection is present in SC, but not EC/FC. All calculated percentages were relative to the number of connections present either in SC and/or EC/FC. For FC, connections were thresholded using the null distribution in section Threshold From Null Distribution and the mean FC network was derived by taking only connections existing in at least half of the subjects. Since the network densities of EC and FC do not agree, an additional analysis was performed using custom thresholds on the mirrored EC and FC networks, set to yield a 10% false positive rate of connections present in EC/FC but not SC.

As the temporal resolution given by the repetition time (TR) was much higher than in conventional fMRI (1–3 s), the influence of the TR was also analyzed by downsampling the time series to TRs between 0.1 and 3 s.

Finally, the default-mode network (DMN) was analyzed as an example of a well-studied network in literature. The DMN consists of three main brain areas in each hemisphere: the medial prefrontal cortex (mPFC), the inferior parietal cortex (IPC), and the posterior cingulate cortex (PCC). Furthermore, the hippocampus (HIP) and the temporal cortex (TC) are also sometimes included in the network. Note that the network estimation was still performed at the whole-brain level, after which only the connections within the DMN were examined in more detail.

## Results

### Simulation study

**Figure 1:**
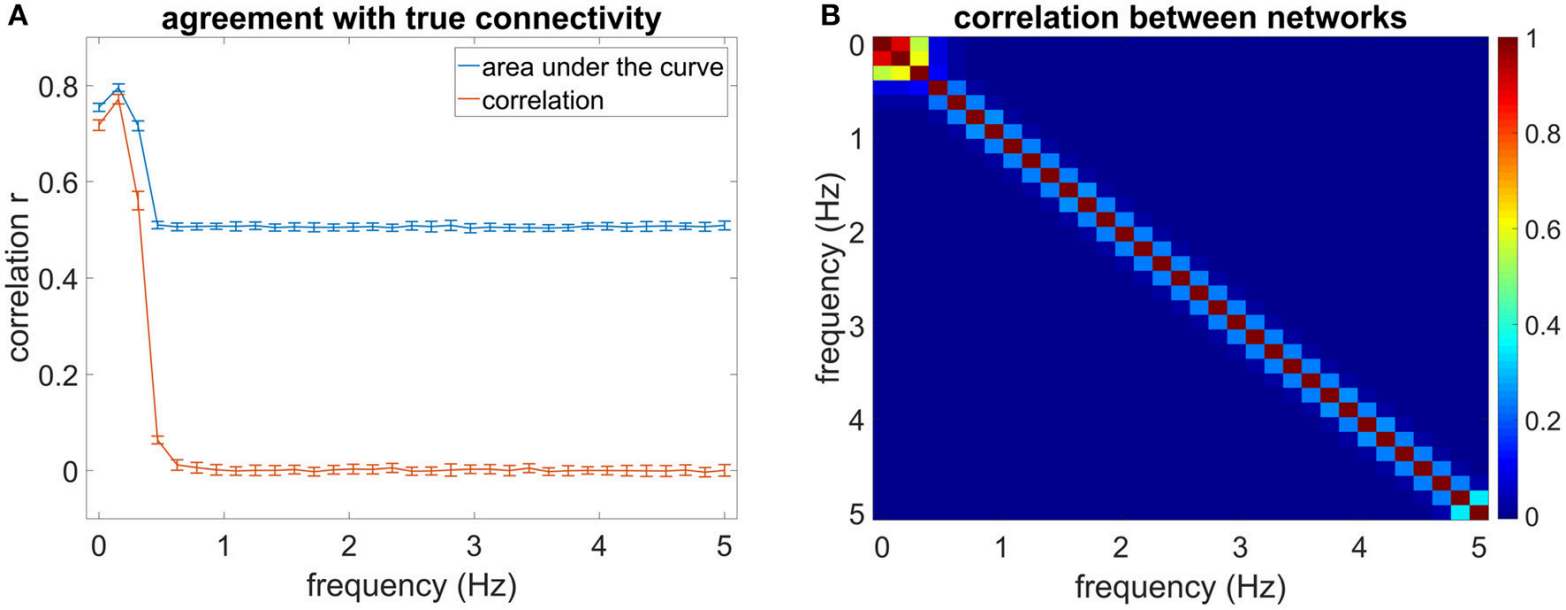
**(A)** Shows the mean correlation of the estimated connectivity matrices and the true connectivity and the area under the ROC-curve for 20 data sets at different frequencies. The error bars show the standard deviation of the estimated correlation or AUC values over data sets. **(B)** Shows the correlation between estimated networks at different frequencies.

For the simulated data, only the low-frequency bins were analyzed as the higher frequency bins contained mainly noise due to the convolution with the canonical HRF. This left three frequency bins with 0 < *f* < 0.45 *Hz* (cf. Figure 1).

#### Influence of length of time series and SNR

**Figure 2:**
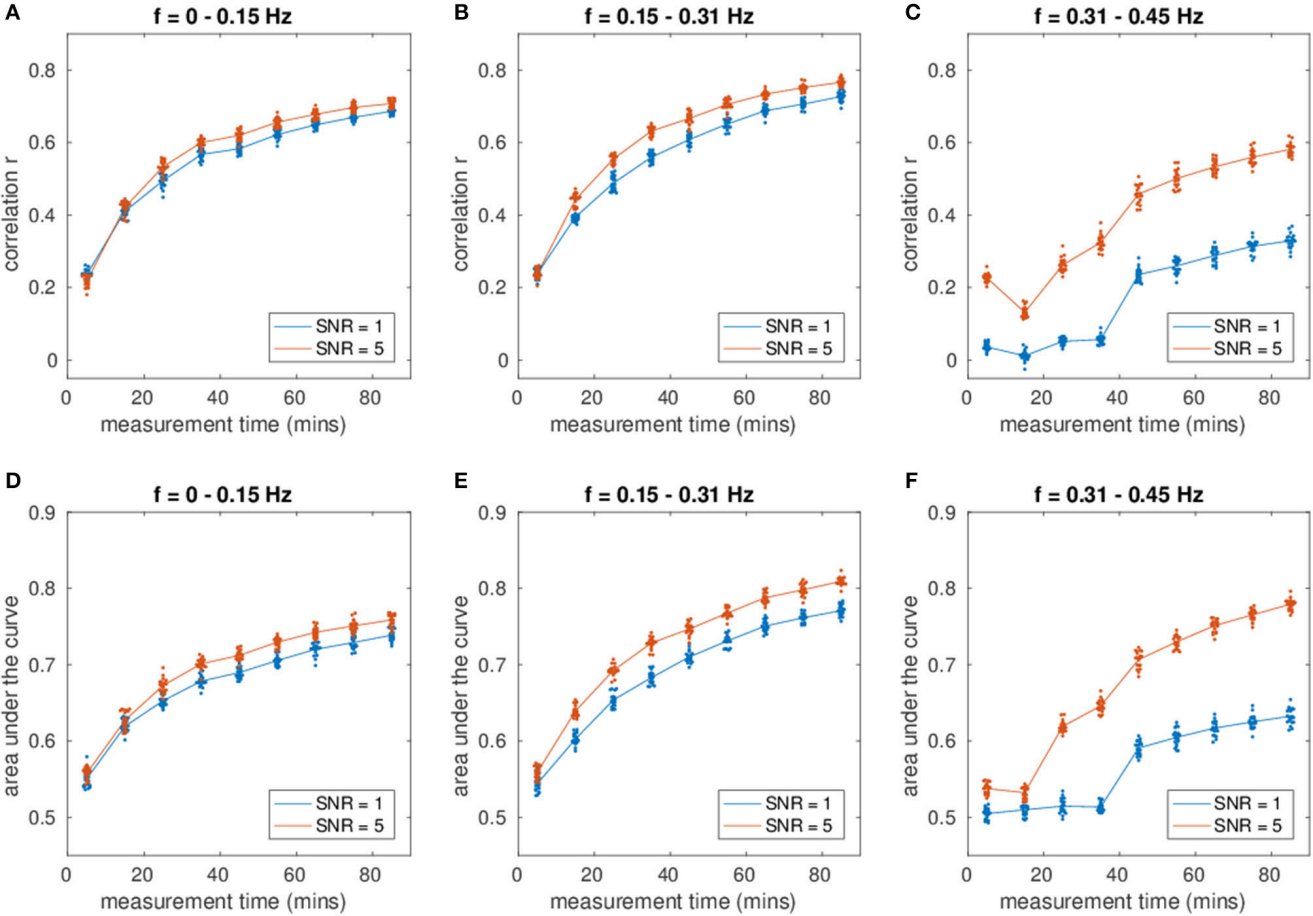
**(A–C)** Shows the correlation of the estimated connectivity matrices and the true connectivity with varying dataset length. **(D–F)** Shows the area under the ROC-curve (AUC) for the different lengths. Different colors correspond to different SNR values.

Figures 2A–C show the mean correlation of the estimated network with the true network for the first three frequency bins according to measurement time and SNR. Figures 2D–F show the mean AUC values for the first three frequency bins. The correlation and AUC increases monotonically with increasing length of the time series. Moreover, an increase in SNR improves the estimation.

With increasing measurement time, the correlation of the networks and the AUC increases strongly. After a measurement time of ~35 min the slope of the correlation and AUC plot is shallower. Most of the estimation power is concentrated in the frequency range between 0 and 0.31 Hz, yielding higher correlation of the estimated networks with the true networks than the higher frequency bins (see Figure 1). For the first frequency bin, the correlation reaches a level of close to 0.8 for an SNR of 5. For the second frequency bin (0.15–0.31 Hz), the correlation and AUC even go beyond 0.8 for high SNR. For the third frequency bin, the correlation ranges for a measurement time of 85 min between 0.4 for the lower SNR and 0.75 for the higher SNR. The AUC varies between 0.6 and 0.75, where a value of 0.5 equals pure chance.

The SNR has a strong influence on the estimation: An increase of SNR improves the estimation. Higher frequencies are especially sensitive to measurement time and SNR (cf. 2 C/F). While for a SNR of 5 the estimation is still quite good, especially for long measurements of 40 min and more, the estimation for the lower SNR declines for measurements shorter than approximately 45 min. At shorter measurements the noise predominates in the CSD. However, at approximately 45 min there is a prominent jump in the correlation and AUC values. At such long measurement times, sufficient noise averaging occurs and the true covariance structure can be retrieved fairly well.

#### Influence of type of noise

Table 1 shows results from the networks simulated using different noise types, averaged over the three low-frequency bins.

**Table 1:** Influence of type of noise.

**Noise type\SNR**	**1**	**5**
**LENGTH OF TS: 20,000 DATA POINTS**		
White	0.462 ± 0.003	0.588 ± 0.003
Mixture	0.414 ± 0.006	0.565 ± 0.004
Temporally correlated	0.410 ± 0.006	0.562 ± 0.005
**LENGTH OF TS: 40,000 DATA POINTS**		
White	0.558 ± 0.003	0.710 ± 0.003
Mixture	0.505 ± 0.002	0.686 ± 0.003
Temporally correlated	0.499 ± 0.001	0.682 ± 0.004

*Mean correlation over 20 datasets and the first three frequency bins for different noise compositions, SNRs and different length of measurement time*.

At low SNR, the differences for the various types of noise are more prominent than at high SNR, where the differences start to vanish. The difference between the mixture of white and temporal noise and pure temporal noise, however, is not so prominent. An increase of measurement time improves the estimation itself, but does not have an influence on the observed differences between noise types.

#### Influence of missing nodes on the estimation

**Figure 3:**
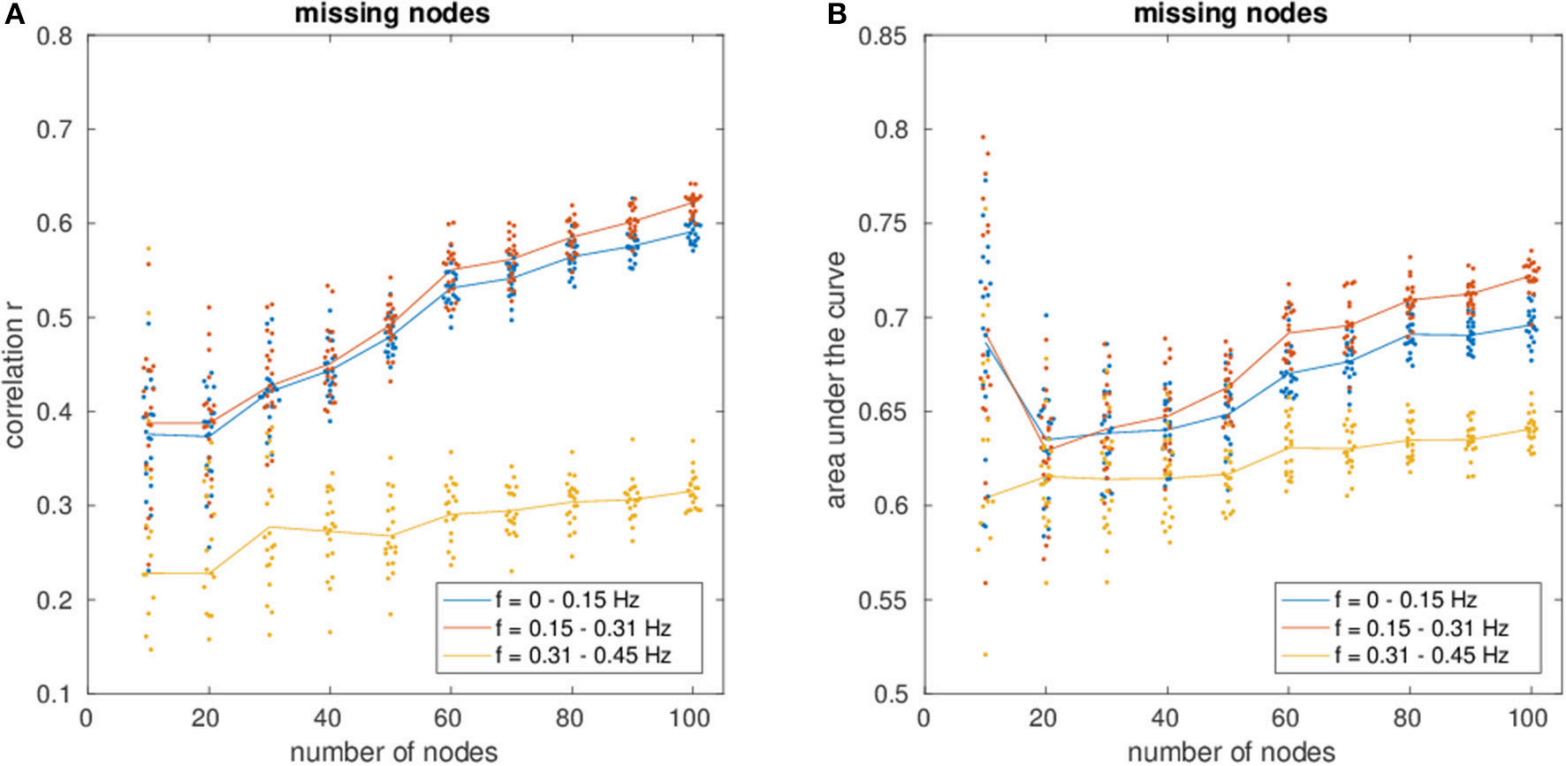
**(A)** Shows the correlation of the estimated connectivity matrices and the true connectivity as a function of varying network size with missing nodes present. **(B)** Shows the corresponding area under the ROC-curve (AUC). Different colors correspond to different frequency bins.

Figure 3 shows an increase of estimation power with increasing fraction of observed nodes (Figure 3A, correlation and Figure 3B, AUC), indicating the importance of the missing nodes on the network.

Regarding the variance of the estimation for different network sizes, a strong decrease in variance with decreasing fraction of missing nodes can be observed, demonstrating the beneficial influence of more nodes and therefore more information to recover the network.

#### Influence of hemodynamic variability

**Figure 4:**
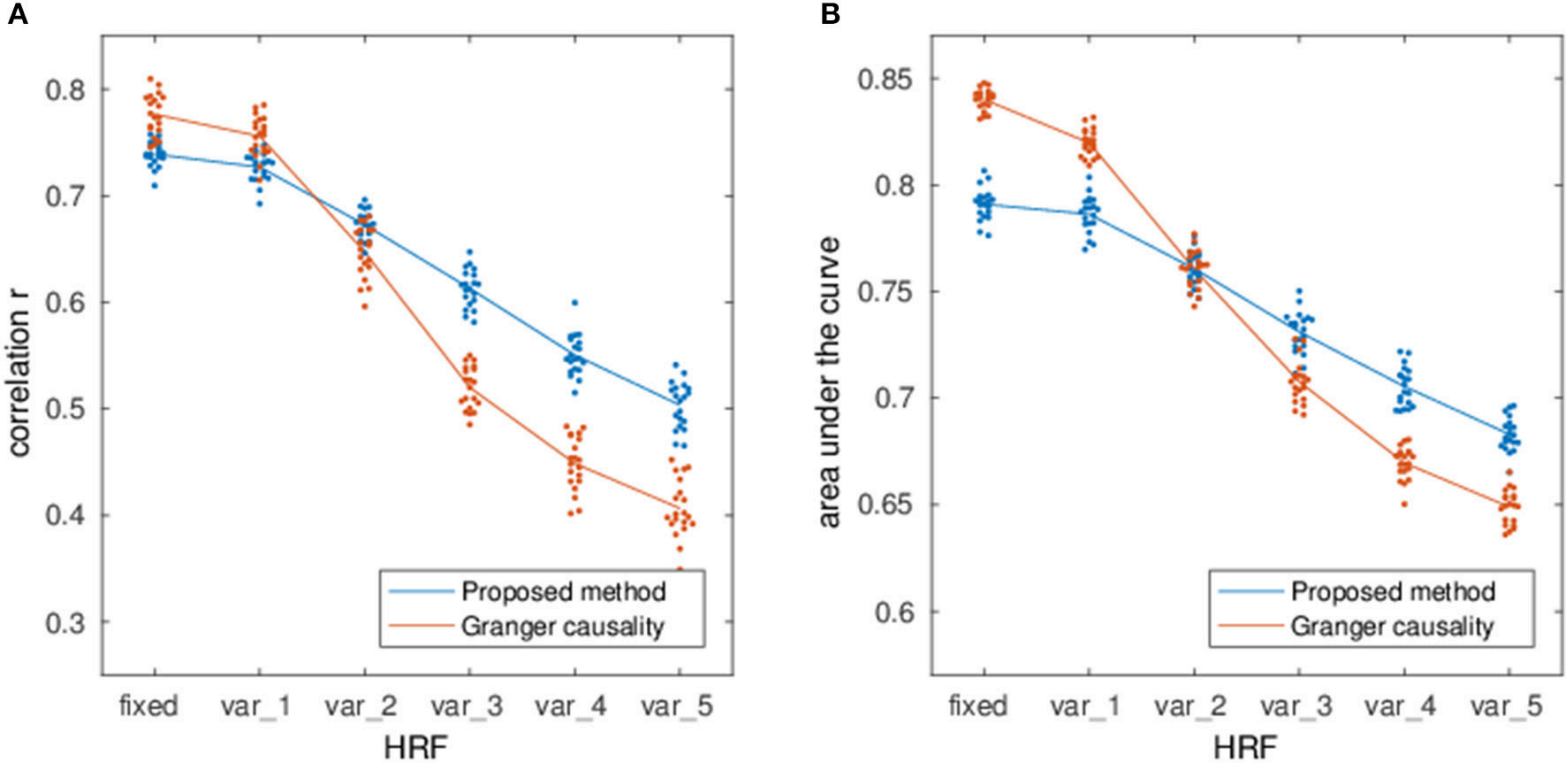
Correlation **(A)** and AUC **(B)** of the EC estimation method as a function of HRF variability (blue line). The labels var_*k* denote simulations performed with double-gamma HRFs where the onset of the gamma functions was randomly varied between ±*k* seconds and the dispersion and amplitude parameters were randomly varied by a factor of up to *k* with respect to the canonical HRF. For comparison, the red line shows the performance of multivariate Granger Causality estimation.

Figure 4 shows the estimation performance under various degrees of HRF variability as well as a comparison with multivariate GC (Barnett and Seth, 2014). While both methods perform well with fixed HRFs, they also show a clear degradation under variable HRF conditions, although GC is more susceptible to the confounding influence of the HRF on temporal precedence information.

#### Mean network from all data sets

**Figure 5:**
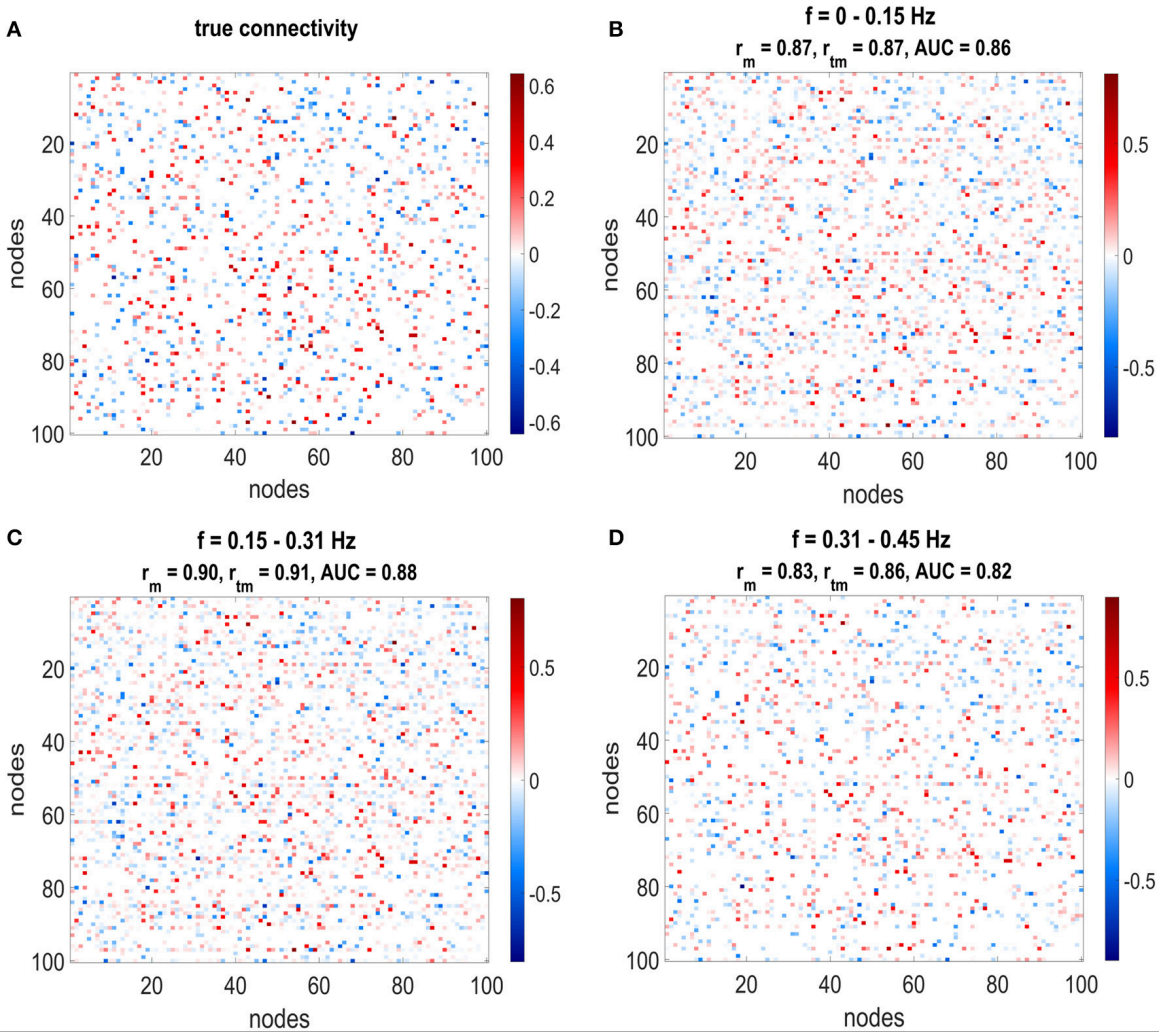
The estimated connectivity matrices and the true connectivity for three different frequency ranges are depicted. **(A)** Shows the true connectivity used to simulate the data, **(B–D)** Shows the estimated connectivity matrices for the different frequency ranges. The plots show the weighted adjacency matrices of the network, where red entries stand for connections with positive weights, blue entries for connections with negative weights and white depicts no connection. Furthermore, the hue of the color depicts the strength of the connection. Correlation and AUC values are given in the title of each plot: *r*_*m*_ stands for correlation with mean network without threshold from null distribution, *r*_*tm*_ corresponds to correlation with mean network with threshold from null distribution. All correlations and AUC values are above 0.8.

Figure 5 shows the comparison between the true network (Figure 5A) and the mean estimated network (Figures 5B–D) for the lowest three frequency bins. Each entry in the matrix plot corresponds to a directed connection, where the connection goes from column to line. Red entries in the matrix plot correspond to positive (excitatory) connections and blue ones to negative (inhibitory) connections. Moreover, the hue of the color depicts the strength of the connections. Due to the random nature of the simulations, there is no structure in the network that may facilitate visual inspection, but it can still be observed that strong connections are especially well estimated.

The titles from Figures 5B–D show the correlation and AUC values for the mean networks without thresholding by the null distribution (AUC and *r*_*m*_) and correlation with applied thresholds to exclude non-significant connections (*r*_*tm*_). Taking solely the mean over all networks yields high correlations between 0.8 and 0.9 for the first three frequency bins. By removing the non-significant connections from the network using the threshold from the null distribution and taking only connections which exist in at least 50% of the estimated networks increases the correlation even further. The area under the ROC curve lies between 0.8 and 0.9.

### Real fMRI data

**Figure 6:**
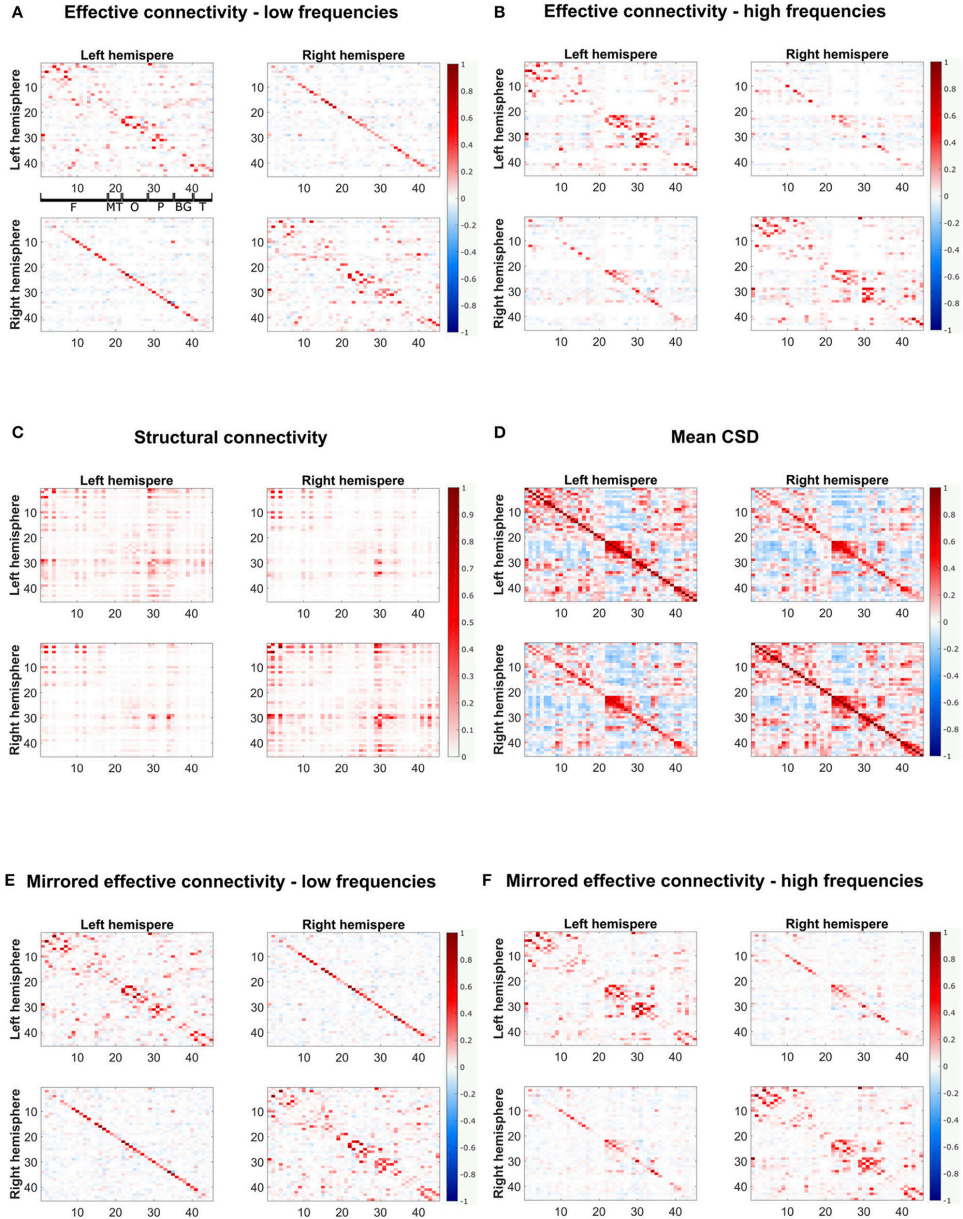
The estimated effective connectivity matrices from fMRI data for low- and high-frequency bands and their mirrored networks and the structural connectivity from DTI are depicted. **(A,B)** Show the effective connectivity for low- and high-frequency bands, respectively, **(E,F)** Show the mirrored estimated connectivity matrices for the different frequency ranges. For visualization purposes, only connections present in at least two thirds of frequencies in the respective frequency bands are shown. **(C)** Shows the structural connectivity and **(D)** the mean CSD over all subjects and frequencies. The plots show the weighted adjacency matrices of the network, where red entries stand for positive connections, blue entries for negative connections and white depicts no connection. Furthermore, the color hue depicts the strength of the connections. The axes refer to indices from the AAL atlas, separated between left and right hemispheres, corresponding to the regions indicated in the topmost left plot: F, frontal; MT, mesial temporal; O, occipital; P, parietal; BG, basal ganglia; T, temporal.

Applying the threshold from the null distribution and deriving the confidence intervals from the bootstrapped networks, sparse networks were achieved in the real fMRI data (see Figure 6). We separately consider a low frequency band (0–0.31 Hz) and a high frequency band (0.31–5 Hz). The estimated average low and high frequency networks are quite similar (*r* = 0.6) and, moreover show a strong similarity between hemispheres (*r* = 0.8 for the low frequency network, *r* = 0.9 for high frequencies).

The results from the analysis of the variability of the SC and EC networks over subjects can be seen in Table 2. EC shows strong variability both for low and high frequencies with a correlation around 0.2–0.3 and AUC of around 0.6. SC, however, shows a strong agreement between subjects (*r* = 0.85 and AUC = 0.80), showing the high stability of the SC across subjects.

**Table 2:** Variability of networks between subjects.

	**Correlation r**	**AUC of ROC**
Low-frequency band	0.228 ± 0.054	0.552 ± 0.022
High-frequency band	0.256 ± 0.060	0.637 ± 0.035
DTI	0.845 ± 0.049	0.804 ± 0.042

*Correlation and AUC values (mean and standard deviations) calculated between estimated networks from fMRI and structural connectivity from DTI between subjects*.

#### Comparison with DTI tractography

Due to the lack of a gold standard the estimated EC networks were compared to SC from DTI. The resulting correlation and AUC values are displayed in Table 3.

**Table 3:** Agreement between EC/FC and SC.

	**Correlation r**	**AUC of ROC**
**LOW FREQUENCY**		
*G*+*G*^*T*^	0.26	0.55
*G*	0.24	0.56
**HIGH FREQUENCY**		
*G*+*G*^*T*^	0.43	0.62
*G*	0.41	0.60
**MEAN CSD**		
CSD	0.18	0.56

*Correlation and AUC values between mean estimated effective connectivity networks and functional connectivity networks from fMRI and structural connectivity from DTI*.

The low frequency network shows some correlation with SC (*r* = 0.24 and AUC = 0.56), which is only slightly increased for the mirrored network (*r* = 0.26 and AUC = 0.55). For the high frequency band the agreement between SC and EC is much more pronounced. For the mirrored network we have a correlation of *r* = 0.43 and even for the normal EC the correlation is quite high with 0.41 (cf. Table 3). However, there are still clear discordances, as seen in the AUC values of 0.62 for the mirrored and 0.60 for the normal EC.

Figure 6 shows both normal and mirrored estimated EC networks for the low- and the high-frequency band (Figures 6A,B,E,F), the SC network (Figure 6C) and the mean CSD over all subjects and frequencies (Figure 6D). In agreement with SC, the EC shows strong connectivity in the frontal and and parietal regions. Moreover, the lack of connections between frontal and mesial temporal and occipital regions is correctly identified. Discrepancies are mainly visible along the diagonals of the top-right and bottom-left quadrants corresponding to interhemispheric connections between homologous regions. Differences are also seen close the diagonal of the top-left and bottom-right quadrants because local short-range connections are not easily recovered by diffusion tractography. The high frequency EC network, however, has less pronounced interhemispheric connections, therefore also yielding higher correlation with SC.

#### Comparison with raw cross-spectral density

The results of EC were also compared to standard functional connectivity, represented by the cross spectral density (Figure 6D). Mean CSD shows lower agreement with SC (*r* = 0.18 and AUC = 0.56). It can be seen that the EC networks are much sparser than the CSD functional network. Furthermore, the block of massive inter-hemispheric connections in the occipital lobe present in the CSD (between regions 20–30 on the top-right and bottom-left quadrants) vanished in the low frequency EC network and is very much reduced in the high frequency EC network.

**Figure 7:**
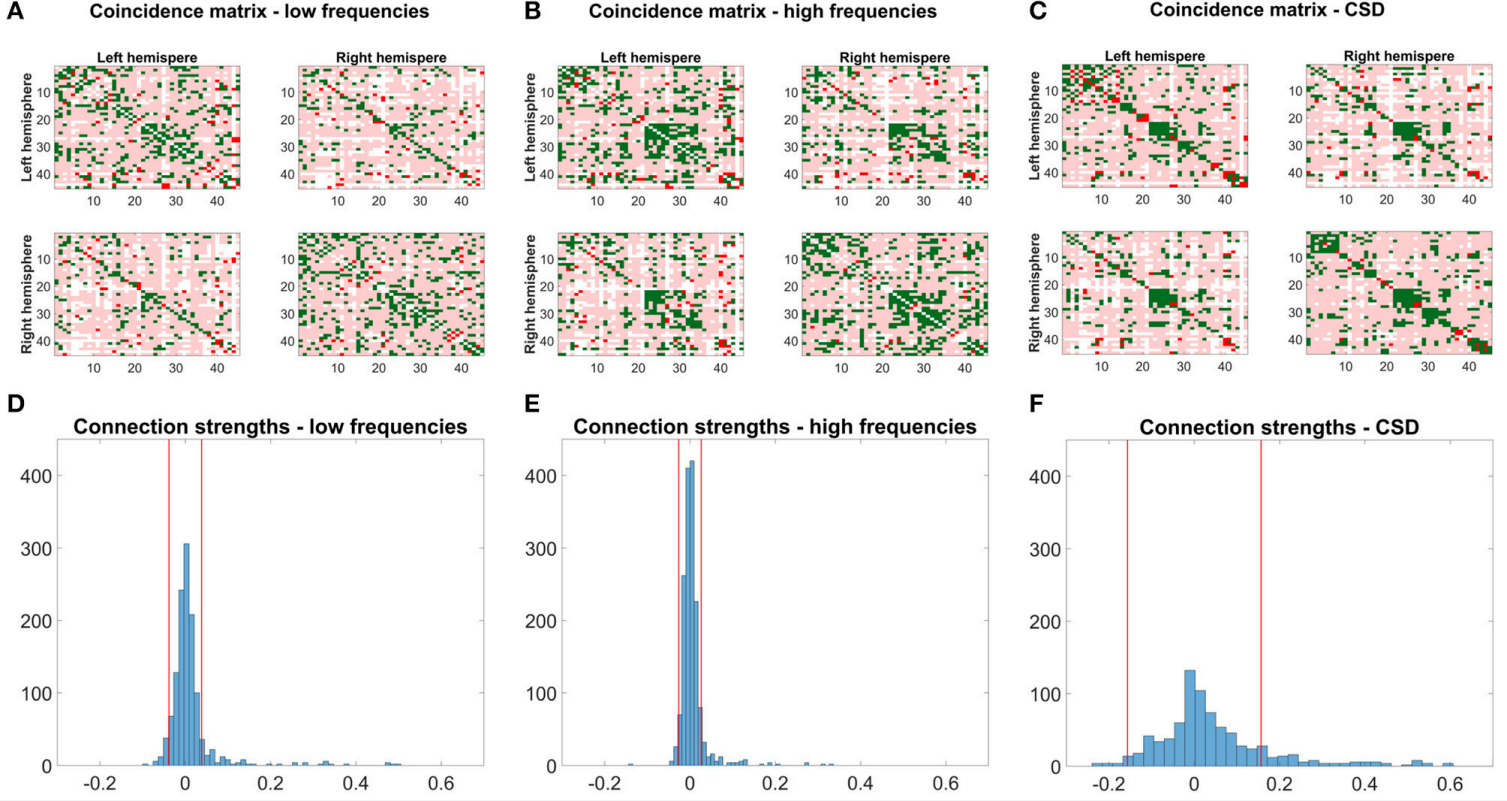
Coincidence maps between structural connectivity from DTI and the mirrored low- **(A)** and high- **(B)** frequency networks and the cross-spectral density **(C)**. EC and FC networks were thresholded to yield a false positive rate (FPR) of 10%. Green entries depict connections existing in SC and EC/FC, bright red entries depict connections only existing in EC/FC. Pale red entries denote connections present in SC but not in EC/FC. **(D–F)** Shows a histogram of the connection strengths for connections not present in SC. The red lines depict the threshold chosen to obtain a FPR of 10%.

Figures 7A–C shows coincidence maps, where green entries depict agreement between SC and EC/FC, bright red entries connections that are only present in EC/FC but not SC, and pale red entries connections that are only present in SC but not in EC/FC. For comparison purposes, the EC/FC networks were thresholded to have a false positive rate (FPR) of 10% to gain an insight into the agreement of SC with EC/FC given a fixed FPR. At this FPR, many SC connections are not reflected in EC/FC. Nevertheless, the high-frequency EC shows the highest agreement with SC (Figure 7B). Low-frequency EC and FC show similar agreement (cf. Table 4), although for low-frequency EC the agreeing connections are very scattered while for FC the agreeing connections tend to form clusters.

**Table 4:** Coincidence of connections in SC and EC/FC.

	**Connections in SC and EC/FC(%)**	**Connections in EC/FC, not SC(%)**	**Connections in SC, not EC/FC(%)**	**Network density EC/FC(%)**	**Network density SC(%)**
Low frequencies	60.6	15.5 (9.9)	22.7	75.7 (43.6)	78
High frequencies	77.1	20.6 (11.6)	2.28	95.1 (49.9)	78
CSD	55.8	11.9	32.3	59.9	78
Low frequencies–adapted FPR	18.5	2.8	77.8	17.1	78
High frequencies–adapted FPR	25.0	2.8	71.3	22.3	78
CSD-adapted FPR	18.6	2.7	78.7	17.1	78

*Percentage of connections agreeing between SC and EC/FC. EC networks are mirrored; values for non-mirrored EC are given in brackets. In the last three rows, the percentages of agreeing connections are shown for mirrored EC and FC whose false positive rate (FPR) was adapted to 10% for a better comparison*.

Figures 7D–F shows histograms of the connection strengths for connections not present in SC but in EC/FC. Such false connections (without an underlying structural basis) for low- and high-frequency EC networks are very weak even though they were statistically significant. In contrast, the connection strengths of the false connections for CSD range from −0.3 up to 0.6.

To quantify the agreement between SC and EC/FC, the percentage of connections agreeing between SC and EC/FC, connections only present in EC/FC, and connections only present in SC were calculated (see Table 4). For non-adapted network densities, SC and EC correspond better than CSD, which is mainly due to the higher number of connections in EC than FC. For connections present in EC/FC, but not in SC, the percentage is similar for normal EC and SC. Looking at the networks with adapted densities, mirrored EC for high frequencies and SC have the highest agreement; FC and EC for low frequencies have similar but lower agreement with SC. However, as seen in Figures 7D–F, “wrong” connections, which are present in EC/FC but not SC, cover a much broader range for FC than for EC.

**Figure 8:**
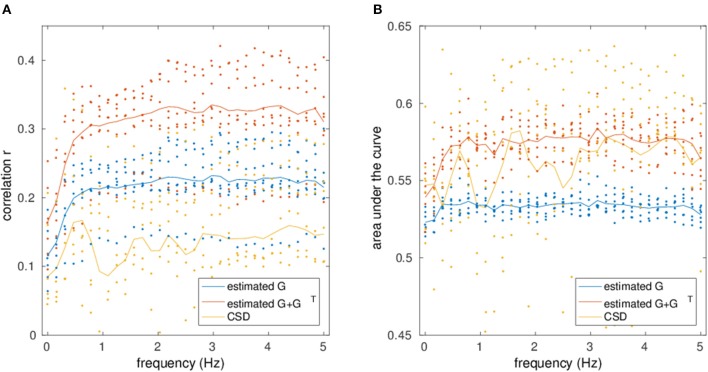
Correlation **(A)** and AUC **(B)** values for the comparison between the estimated networks from fMRI/ CSD matrices and structural connectivity from DTI is shown at different frequencies.

Figure 8 depicts the mean correlation and AUC values of all subjects over all frequencies of EC/FC with SC from DTI. The correlation and AUC is always higher for mirrored EC networks than for normal EC networks. The correlation is also higher when compared to the raw CSD. However, for the AUC there is high variability and strong overlap between mirrored EC and CSD, although mirrored EC is still mostly above CSD.

This can also be seen in Table 5, where the statistical significance of the *t*-test of EC correlation and AUC values vs. those from CSD are tested, where correlation values were z-transformed prior to the *t*-test. For the correlation at low frequencies, mirrored EC is better than CSD, but other measures are not significantly different. However, at high frequencies, both normal and mirrored EC correlate significantly better than CSD, but only the mirrored EC shows significantly higher AUC than the CSD.

**Table 5:** Significance of agreement between EC/FC and SC.

	**Correlation r**		**AUC of ROC**	
**Test**	**Significant**	***p*-value**	**Significant**	***p*-value**
**LOW FREQUENCY**				
*G*+*G*^*T*^ vs. CSD	1	0.01	0	0.79
*G* vs. *CSD*	0	0.84	0	0.24
**HIGH FREQUENCY**				
*G*+*G*^*T*^ vs. CSD	1	<0.001	1	0.02
*G* vs. *CSD*	1	<0.001	0	0.15

*Test for significance between estimated EC networks from fMRI and pure cross-spectral density matrices. The correlation and AUC values between estimated networks/CSD and SC from DTI were used and the mean value calculated for the high and the low frequency band for each subject*.

#### Influence of TR/sampling rate

**Figure 9:**
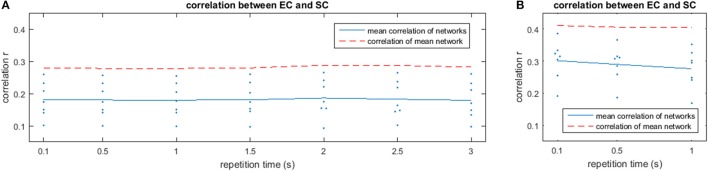
Correlation values for the comparison between the estimated networks from fMRI matrices and structural connectivity from DTI is shown for different repetition times (TR) in the low-frequency **(A)** and high-frequency **(B)** bands. The red dashed line shows the correlation of the mean network over subjects with the structural connectivity from DTI.

The results of the analysis with different TRs are depicted in Figure 9 for low frequencies (A) and high frequencies (B). While low frequencies are available at all examined TRs, high frequencies could only be analyzed at shorter TRs. Each plot shows the mean correlation of the estimated mirrored EC with SC for different TRs (blue solid line) and correlation of the mean network over all subjects with SC (red dashed line). While the variability between subjects is quite high, the estimated network barely changes with decreasing TR, which is portrayed by a basically horizontal line for the correlation across different repetition times, indicating that the method is also appropriate at slower TRs. Nevertheless, the higher agreement between EC and SC found at higher frequencies can only be observed at TRs sufficiently short to observe such frequencies.

#### Default-mode network

**Figure 10:**
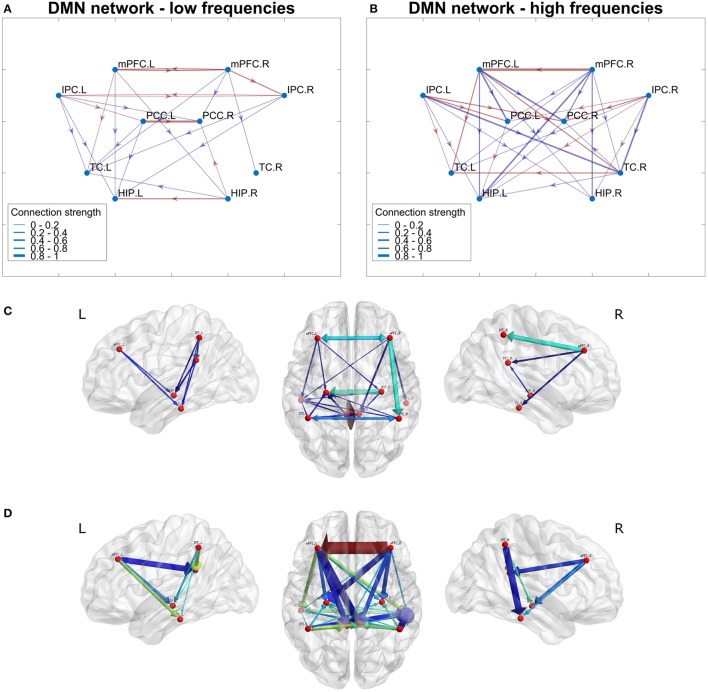
Networks for low- and high-frequency bands of the default-mode network (DMN). In **(A,B)**, network connections are depicted by lines between regions. Red lines correspond to positive connections and blue lines to negative connections. Connection strength ranges are illustrated by the line thickness. The connection strengths for both networks are normalized to the strongest connection in the network. **(C,D)** Show the same networks (low frequencies **C**, high frequencies **D**) where the network is projected onto a generic brain. Both color and arrow thickness represents absolute connection strengths (The brain networks in **(C,D)** were visualized with BrainNet Viewer (http://www.nitrc.org/projects/bnv/) (Xia et al., 2013).

From the estimated networks, the default-mode network was examined more closely. In Figure 10, the DMN is presented for both the low- and high-frequency bands. In Figures 10A,B, the low- and high-frequency band networks are shown with dots for the brain regions connected by red (positive weights) and blue (negative weights) lines. The width of the lines is proportional to the connection strength. As both networks are normalized to their respective strongest connection, the line thickness give only relative information about connection strength and cannot be compared directly across frequency bands.

For the low-frequency band the homologous brain areas were much more connected than for the high-frequency band. In the low-frequency network, all regions except TC were connected to their homologous regions. At high frequencies, only TC and mPFC had connections between homologous brain regions. Furthermore, the low-frequency network was much sparser, while the high-frequency network showed many quite strong connections, which were ordered in a symmetric fashion. For both networks, mPFC projects to other regions but does not receive input except from its homologous region in the other hemisphere. Also IPC only projects to other regions; however, it receives input from its homologous region only at low frequencies. PCC both receives input from mPFC and IPC and projects further to HIP. The TC mainly receives input but for high frequencies it also projects to PCC.

## Discussion

We presented a method to estimate the effective connectivity from fMRI data, based on the symmetric cross-spectral density matrix of the acquired time series. As many different topologies can give rise to the same cross-spectral structure, the ambiguity in the estimation is resolved by using a L1-regularization preferring sparse networks. This is also a popular assumption in the GC framework, particularly in the case of voxel-wise connectivity estimation, due to the resulting large number of network nodes (Valdés-Sosa et al., 2005; Haufe et al., 2010; Garg et al., 2011; Tang et al., 2012), and is supported by the observation that cerebral connections tend to be highly selective (Valdés-Sosa et al., 2005; Sanchez-Bornot et al., 2008).

### Simulated data

As a proof of principle, the method was first applied to simulated data, where the influence of several parameters was analyzed.

Considering the length of the dataset and the SNR, increasing the SNR improved the estimation. An increase in the length of the time series also improved the estimation considerably up to a measurement time of 40 min, after which the increase became slower (cf. Figure 2). Although the methodology only depends on the cross-spectral density and not directly on the time course of the neuronal activity, increasing the length of the time series yields better estimates of the sample CSD. Furthermore, increasing the length of the time series allows for a finer frequency resolution of the CSD. However, long measurements might be problematic from the point of view of subject comfort and motion artifacts. Moreover, this assumes data stationarity, which is questionable for long measurements, particularly given the prevalence of dynamic connectivity states (Calhoun et al., 2014; Preti et al., 2016). Hence, a possible solution would be the acquisition of several shorter measurements and taking the mean cross-spectrum over the measurements and over connectivity states.

To further analyse the influence of the type of noise on the estimation, EC was estimated for pure white noise, pure pink noise and a mixture of both for different length of time series and SNR. Pure pink noise and the mixture gave similar results, mainly due to the high degree of pink noise in the mixed noise. For increasing SNR, the difference in estimation power for white, pink or mixed noise decreases, giving good results also for more realistic noise. But even for a lower SNR of 1, the difference is not very striking (cf. Table 1). The loss of estimation power for temporal noise is due to the correlation between adjacent data points, which leads to a loss of degrees of freedom and therefore a loss of information in the CSD. However, the connectivity information can nonetheless be retrieved from the data unless data sets are very short and with high temporal autocorrelation. For higher SNR the information from the true signal dominates the data, leading to better estimation results.

Often, effective connectivity analyses will only be performed on a small number of brain nodes of interest. This, however, poses the problem of missing nodes in the estimation of networks, which might lead to an erroneous estimation of the connectivity (Eichler, 2005; Daunizeau et al., 2011; Waldorp et al., 2011). For example, an indirect influence from a missing node on two nodes of interest might be interpreted as a spurious link between those two nodes. Therefore, the proposed method was applied to various network sizes that were part of larger networks with unobserved nodes. Not surprisingly, the estimation power increases monotonically with increasing fraction of observed nodes of the network (cf. Figure 3). This demonstrates the importance of minimizing the number of missing nodes. As fMRI data sets are usually whole brain scans and the computational efficiency of the proposed method allows for a high number of nodes (estimation time of a few minutes for a network with a hundred nodes on a standard computer), this problem can be overcome by estimating the full network and retrieving the partial network of interest afterwards.

The reduced dependence on HRF variability in comparison to lag-based Granger causality (cf. Figure 4) is especially relevant for fMRI given the indirect nature of the measured hemodynamic signals. This further confirms previous results on the possibility to estimate sparse networks from lag-free covariances (Pernice and Rotter, 2013; Schiefer et al., 2018). Nevertheless, it is clear that hemodynamic variability still acts as an important confounder on observed time-series correlations, so that integrating hemodynamic information, either via separate HRF estimates (Wu et al., 2013; Proulx et al., 2014) or by specifically including hemodynamics in the generative model (Ryali et al., 2011; Friston et al., 2014b) would be beneficial to EC estimation.

All in all, the analysis of the proposed method on simulated data proved quite successful, showing high agreement between the estimated and the true networks (cf. Figure 5). This might in part be due to the method used to generate the simulated data, which was closely matched to the estimation model. However, Pernice and Rotter (2013) also demonstrated good results in the estimation of networks of leaky integrate-and-fire neurons, suggesting that the analysis is applicable to a wide variety of different data types.

### fMRI data

In a second step, the proposed method was applied to fast fMRI data. Correlating the estimated networks over frequencies suggested a clustering in two frequency bands: A low-frequency band from *f* = 0–0.31 Hz and a high-frequency band *f* = 0.31–5 Hz. Interestingly, high-frequency BOLD signal fluctuations above 0.1−0.2 Hz have rarely been considered in conventional functional connectivity analyses. However, recently emerged fast fMRI sequences allow to analyse such higher frequencies, with multiple studies suggesting that they contain relevant information (Lee et al., 2013; Yuan et al., 2014; Trapp et al., 2017). High-frequency connectivity could not be examined in our simulations as it was completely attenuated by the convolution with the canonical HRF, but it has been recently reported that resting-state fMRI may be driven by narrower HRFs with non-negligible contributions at high frequencies (Chen and Glover, 2015). This is also in line with another recent study that found that information in Granger causality estimates is carried at frequencies up to 3 Hz in fMRI data (Lin et al., 2015).

The estimated networks showed a strong similarity between hemispheres (*r* = 0.8 for the low frequency network, *r* = 0.9 for high frequencies), which would be expected. Both networks showed strong intra-hemispheric connections in the frontal, occipital and parietal lobes (see Figure 6). Although the networks for low- and high-frequencies were similar, the low-frequency network was much sparser and less symmetric than the high-frequency network. Moreover, the low-frequency network showed strong inter-hemispheric connections between homologous brain regions in both hemispheres, which is less pronounced in the high-frequency network.

Due to the lack of gold standard, the estimated effective connectivity networks were compared to structural connectivity from DTI. While the SC networks were very stable over subjects, the EC networks showed quite high variability. This is not very surprising, since the SC network corresponds to the “hard wiring” of the brain, which is expected to be similar for different individuals. The EC, however, is estimated from resting-state data. Although some general patterns evolve in resting-state data, the processing network might vary strongly between subjects (Mueller et al., 2013). Furthermore, during the measurement, subjects might not always be in perfect resting state, but might let their mind wander leading to an altered network (Kucyi, 2017).

The comparison between EC and SC yielded relatively low correlation values, which could be partly attributed to the symmetry of undirected SC measures. The correlation increased significantly after mirroring the estimated EC. The agreement between the high-frequency network and SC was significantly higher than for the low-frequency network. In part this is due to the presence of strong inter-hemispheric connections between almost all homologous brain regions in the low-frequency band, which is a typical point of discordance with SC (Messé et al., 2014). For the high-frequency band, however, these inter-hemispheric connections were less pronounced yielding much higher agreement between EC and SC. Furthermore, the low-frequency network is much sparser than both the high-frequency network and SC, hinting to the idea that for low frequencies much fewer structural connections are active than for high frequencies due to relatively short conduction delays between brain areas leading to activity at higher frequencies. We emphasize again, however, that comparison with SC cannot be considered a strict validation, since the static physical connections in SC cannot represent the dynamic connections active in any given time or brain state. Moreover, diffusion tractography itself only provides an imperfect estimate of SC and may miss major structural links, notably interhemispheric connections (Robinson et al., 2014). Nevertheless, consistency with SC can still provide some evidence of a successful EC estimation, especially when considering functional connections not supported by an underlying structural connection. However, we cannot draw firm conclusions on the performance of the method regarding the converse situation, that is, structural connections that may or may not result in an identified functional connection. A true validation of EC in humans would require invasive approaches such as intracranial EEG, which is employed for clinical purposes in some epilepsy patients. Unlike the non-invasive DTI approach used in the current study, intracranial EEG can provide directed measures of effective connectivity (Wendling et al., 2010; Entz et al., 2014) and would be well worth investigating in the future.

As the most widespread technique for functional connectivity analysis is the computation of undirected, potentially band-limited correlations, the proposed method was also compared to the pure cross-spectral density and its agreement with SC. FC was actually better than the normal, unmirrored EC for AUC (*p*_*AUC*_ = 0.02). However, when considering the mirrored EC network, there was significantly better agreement than FC. Moreover, CSD exhibited very high variance both for correlation and AUC compared to normal and mirrored EC. Falsely identified connections (without an underlying structural connection) were very weak for EC but covered a broad range of connectivity strengths for FC (cf. Figure 7). Thus, the estimated EC networks showed more consistency with DTI than the functional networks from CSD. The significance values were however relatively low, which is due to the low number of subjects used to estimate the networks.

As typical fMRI sequences have much lower temporal resolution than the sequence used in this study, the network estimation was performed on datasets that were retrospectively downsampled to longer TRs to analyse the influence of the temporal resolution on the estimation. Correlating the estimated networks with SC showed a relatively stable estimation of the networks even at lower temporal resolutions. The overall results suggest that the measurement length is more important than the number of data points for a given scan time. Note however, that only low-frequency networks can be recovered at long TRs. Higher temporal resolutions was still beneficial for the estimation of high-frequency connectivity (see Figure 9B), which showed better estimation performance than low-frequency networks, as well as preprocessing advantages such as better physiological noise removal (Lin et al., 2012; Jacobs et al., 2014; Korhonen et al., 2014).

Finally, the directed connectivity for the default mode network was retrieved from the estimated network (Figure 10). The low frequency network is much sparser and less symmetrical than the high frequency network. Compared to the results of Miao et al. (2010), who did a Granger causality analysis on the DMN, similar results are obtained, notably the strong connections from all other regions to the PCC. However, differences are also observed in the mPFC where we found mostly outgoing rather than ingoing connections. One potential cause of this discrepancy may be the particular sensitivity of the employed MREG sequence to off-resonances in the mPFC, leading to potential artifacts (Zahneisen et al., 2012; Assländer et al., 2013). Future work will focus on further validation of the inferred directed networks.

## Conclusion

In this paper we presented a method to estimate the effective connectivity from whole brain resting-state fMRI scans from the cross-spectral density in the frequency domain. The influence of different measurement parameters was analyzed in simulated fMRI data, notably showing a reduced dependency on hemodynamic variability compared to lag-based methods such as Granger Causality. The proposed method was further applied to resting-state fMRI data, showing improved consistency with the underlying structural connectivity networks obtained from DTI tractography in comparison to conventional functional connectivity.

## Author contributions

CL, JS, SR, JH, and PL: Conceived and designed the study; CL and JS: Analyzed the data; CL, JS, SR, JH, and PL: Drafted and approved the manuscript.

### Conflict of interest statement

The authors declare that the research was conducted in the absence of any commercial or financial relationships that could be construed as a potential conflict of interest.
